# Supplementary material for: A Case Report of Chronic Stress in Honey Bee Colonies Induced by Pathogens and Acaricide Residues
Source: Pathogens. 2021 Jul 29;10(8):955. doi: 10.3390/pathogens10080955 (PMC8398566; doi:10.3390/pathogens10080955)
Supplement: Supplementary file 1 [file pathogens-10-00955-s001.zip › pathogens-1275517-Supplementary information revised.pdf]

Supplementary information

Table S.1 Details of calculation of TUm values. Total ppb converted to acute dose (µg/bee) considering a maximum consumption rate of 12 mg bb/bee/d (beebread matrix). Surrogate LD50m (µg/bee) calculated by using (Σ pi / LD50i)-1. Ln(TUm: natural logarithm of the toxic unit of the mixture

			Residues						Toxicity (µg/bee)			Toxic Unit of the mixture (TUm)	
			Coumaphos (CMF)		Tau-fluvalinate (FVT)		total	surrogated dose	LD50 coumafos	LD50 tau fluvalinate	surrogate LD50m	TUm	Ln(TUm)
	Matrix	n	ppb	proportion	ppb	proportion	ppb	µg/bee					
**Dead colonies**													
D1		2	$4.35\ 10^{+02}$	$9.84\ 10^{-01}$	7.00	$1.58\ 10^{-02}$	$4.42\ 10^{+02}$	$5.30\ 10^{-03}$			3.14	$1.69\ 10^{-03}$	-6.38
D2		2	$4.15\ 10^{+02}$	9.8310^{-01}	7.00	$1.66\ 10^{-02}$	$4.22\ 10^{+02}$	$5.06\ 10^{-03}$			3.14	$1.61\ 10^{-03}$	-6.43
D3		1	$2.02\ 10^{+02}$	1	<LOQ	0	$2.02\ 10^{+02}$	$2.42\ 10^{-03}$			3.10	$7.82\ 10^{-04}$	-7.15
D4	beebread	2	$3.50\ 10^{+02}$	$9.64\ 10^{-01}$	$1.30\ 10^{+01}$	$3.58\ 10^{-02}$	$3.63\ 10^{+02}$	$4.36\ 10^{-03}$	3.10	$1.22\ 10^{+01}$	3.19	$1.37\ 10^{-03}$	-6.59
D5		2	$3.23\ 10^{+02}$	$9.73\ 10^{-01}$	9.00	$2.71\ 10^{-02}$	$3.32\ 10^{+02}$	$3.98\ 10^{-03}$			3.16	$1.26\ 10^{-03}$	-6.61
mean			$3.45\ 10^{+02}$	$9.81\ 10^{-01}$	7.20	$1.91\ 10^{-02}$	$3.52\ 10^{+02}$	$4.23\ 10^{-03}$			3.14	$1.34\ 10^{-03}$	
d.s			$9.21\ 10^{+01}$	$1.35\ 10-^{02}$	4.71	$1.35\ 10^{-02}$	$9.49\ 10^{+01}$	$1.14\ 10^{-03}$			$3.20\ 10^{-02}$	$3.59\ 10^{-04}$	
**Survivival colonies**													
S1		2	$2.83\ 10^{+02}$	$9.76\ 10^{-01}$	7.00	$2.41\ 10^{-02}$	$2.90\ 10^{+02}$	$3.48\ 10^{-03}$			3.16	$1.10\ 10^{-03}$	-6.81
S2		2	$5.45\ 10^{+02}$	$9.84\ 10^{-01}$	9.00	$1.62\ 10^{-02}$	$5.54\ 10^{+02}$	$6.65\ 10^{-03}$			3.14	$2.12\ 10^{-03}$	-6.16
S3		2	$2.23\ 10^{+03}$	$9.96\ 10^{-01}$	$1.00\ 10^{+01}$	$4.46\ 10^{-03}$	$2.24\ 10^{+03}$	$2.69\ 10^{-02}$			3.11	$8.64\ 10^{-03}$	-4.75
S4		2	$4.65\ 10^{+02}$	$9.69\ 10^{-01}$	$1.50\ 10+^{01}$	$3.13\ 10^{-02}$	$4.80\ 10^{+02}$	$5.76\ 10^{-03}$			3.17	$1.81\ 10^{-03}$	-6.31
S5		2	$1.14\ 10^{+03}$	$9.83\ 10^{-01}$	$2.00\ 10^{+01}$	$1.73\ 10^{-02}$	$1.16\ 10^{+03}$	$1.39\ 10^{-02}$			3.14	$4.41\ 10^{-03}$	-5.42
S6	beebread	2	$3.05\ 10^{+02}$	$9.50\ 10^{-01}$	$1.60\ 10^{+01}$	$4.98\ 10^{-02}$	$3.21\ 10^{+02}$	$3.85\ 10^{-03}$	3.10	$1.22\ 10^{+01}$	3.22	$1.20\ 10^{-03}$	-6.73
S7		2	$7.75\ 10^{+02}$	$9.77\ 10^{-01}$	$1.80\ 10^{+01}$	$2.27\ 10^{-02}$	$7.93\ 10^{+02}$	$9.52\ 10^{-03}$			3.15	$3.02\ 10^{-03}$	-5.80
S8		2	$8.50\ 10^{+02}$	$9.78\ 10^{-01}$	$1.90\ 10^{+01}$	$2.19\ 10^{-02}$	$8.69\ 10^{+02}$	$1.04\ 10^{-02}$			3.15	$3.31\ 10^{-03}$	-5.71
S9		2	$9.36\ 10^{+02}$	$9.86\ 10^{-01}$	$1.30\ 10^{+01}$	$1.37\ 10^{-02}$	$9.49\ 10^{+02}$	$1.14\ 10^{-02}$			3.13	$3.64\ 10^{-03}$	-5.62
S10		2	$8.45\ 10^{+02}$	$9.85\ 10^{-01}$	$1.30\ 10^{+01}$	$1.52\ 10^{-02}$	$8.58\ 10^{+02}$	$1.03\ 10^{-02}$			3.14	$3.28\ 10^{-03}$	-5.72
mean			$8.37\ 10^{+02}$		$1.40\ 10^{+01}$		$8.51\ 10^{+02}$	$1.02\ 10^{-02}$			3.15	$3.25\ 10^{-03}$	
d.s			$5.63\ 10^{+02}$		4.40		$5.63\ 10^{+02}$	$6.76\ 10^{-03}$			$2.95\ 10^{-02}$	$2.18\ 10^{-03}$	
**Overall mean**													
	beebread		$6.73\ 10^{+02}$	$9.83\ 10^{-01}$	$1.17\ 10^{+01}$	$1.71\ 10^{-02}$	$6.85\ 10^{+02}$	$8.22\ 10^{-03}$	3.10	$1.22\ 10^{+01}$	3.14	**$2.62\ 10^{-03}$**	**-5.95**

Table S.2 Primers used for each pathogen in PCR reactions

	Pathogen	Primer	Sequence	Amplicon size
PCR	**N. ceranae**	218 CER-F	5'-CGGCGACGATGTGATATGAAAATATTAA-3'	218-219 pb
		218 CER-R	5'-CCCGGTCATTCTCAAACAAAAAACCG-3'	
	**N.apis**	218 CER-F	5'-GGGGGCATGTCTTTGACGTACTATGTA-3'	321 pb
		218 CER-R	5'-GGGGGGCGTTTAAAATGTGAAACAACTATG-3'	
	**Trypanosomatids**	CRI-SEF	5′- CTTTTGGTCGGTGGAGTGAT- 3′	417 pb
		CRI-SER	5′- GGACGTAATCGGCACAGTTT- 3′	
	**L.passim**	LpCytb_F1	5′-CGAAGTGCACATATATGCTTTAC-3′	247 pb
		LpCytb_R	5′-GCCAAACACCAATAACTGGTACT-3′	
	**C. mellificae**	CmCytb_F	5′–AGTTTGAGCTGTTGGATTTGTT–3′	140 pb
		CmCytb_R	5′–AACCTATTACAGGCACAGTTGC–3′	
	**Neogregarines**	API-NEOF	5′- CCAGCATGGAATAACATGTAAGG- 3′	260 pb
		API-NEOR	5′- GACAGCTTCCAATCTCTAGTCG- 3′	
	**A. woodi**	AW180-FOR	5′-GGAATATGATCTGGTTTAGTTGGTC-3′	180 pb
		AW180-REV	5′- GAATCAATTTCCAAACCCACCAATC-3′	
RT-PCR	**LSV-complex**	LSVdeg-F	5′-GCCWCGRYTGTTGGTYCCCCC-3′	600 pb
		LSVdeg-R	5′-GAGGTGGCGGCGCSAGATAAAGT-3′	
	**AKI**	AKI-F	5′-CTTTCATGATGTGGAAACTCC-3′	100 pb
		AKI-R	5′-AAACTGAATAATACTGTGCGTA-3′	
RT-qPCR	**BQCV**	BQCV 9195F	5′-GGTGCGGGAGATGATATGGA-3′	305 pb
		BQCV 265r	5′-GCCGTCTGAGATGCATGAATAC-3′	
		BQCV 8217T*	5′-FAM-TTTCCATCTTTATCGGTACGCCGCC-TAMRA-3′	
	**DWV**	DWV 9587F	5′-CCTGGACAAGGTCTCGGTAGAA-3′	250 pb
		DWV 9711R	5′-ATTCAGGACCCCACCCAAAT-3′	
		DWV 9627T*	5′-FAM-CATGCTCGAGGATTGGGTCGTCGT-TAMRA-3′	

*5′ (FAM, 6-carboxy-Fluiresceína); 3′ (TAMRA, tetra-metilcarboxyrhodamina)

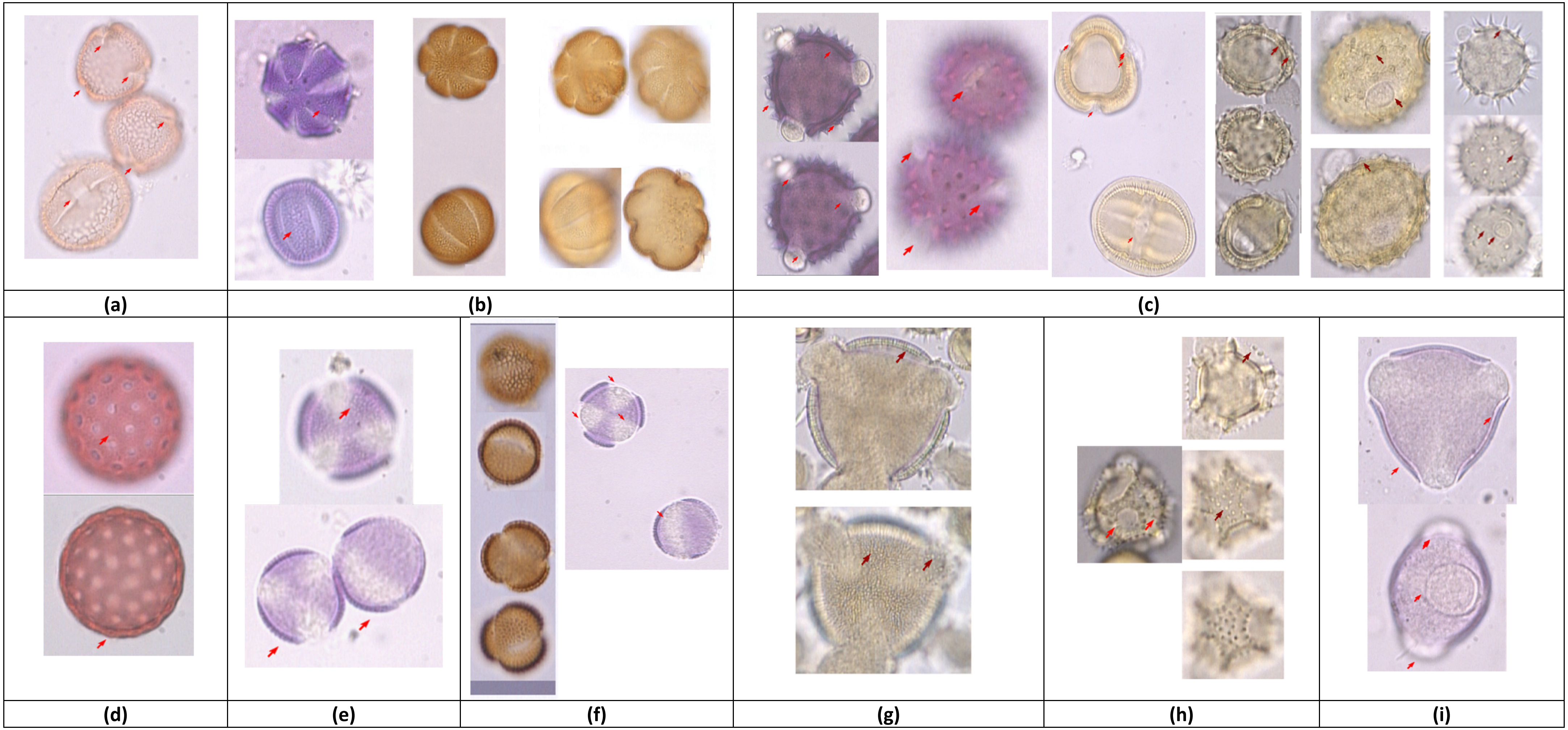

Figure S1. Main taxa identified of the pollen grains found in the beebread samples. Red rows: ornamental features and pollen apertures.
(a) *Hedera helix (Araliaceae*). (b) *Labiatae: Lavandula latifolia* L.; *Thymus* L.; *Rosmarinus officinalis* L. (c) *Asteraceae: Carduus* t.; *Calendula* t.; *Carlina* t.; *Centaurea* t.; *Carthamus lanatus* L; *Helianthus annuus* L. (d) *Chenopodiaceae* . (e) *Brassicaceae: Diplotaxis* t.; (f) *Brassicaceae* t. (g) *Convolvulaceae.* (h) *Cichorioideae*. (i) *Rosaceae*: *Prunnus* t.
